# Characterizing and Visualizing Display and Task Fragmentation in the Electronic Health Record: Mixed Methods Design

**DOI:** 10.2196/18484

**Published:** 2020-10-21

**Authors:** Yalini Senathirajah, David R Kaufman, Kenrick D Cato, Elizabeth M Borycki, Jaime Allen Fawcett, Andre W Kushniruk

**Affiliations:** 1 Department of Biomedical Informatics School of Medicine University of Pittsburgh Pittsburgh, PA United States; 2 Medical Informatics Program School of Health Professions State University of New York - Downstate Health Sciences University Brooklyn, NY United States; 3 School of Nursing Columbia University New York, NY United States; 4 School of Health Information Science University of Victoria Victoria, BC Canada

**Keywords:** electronic health record, electronic medical record, medical informatics, information technology, data visualization, user computer interface

## Abstract

**Background:**

The complexity of health care data and workflow presents challenges to the study of usability in electronic health records (EHRs). Display fragmentation refers to the distribution of relevant data across different screens or otherwise far apart, requiring complex navigation for the user’s workflow. Task and information fragmentation also contribute to cognitive burden.

**Objective:**

This study aims to define and analyze some of the main sources of fragmentation in EHR user interfaces (UIs); discuss relevant theoretical, historical, and practical considerations; and use granular microanalytic methods and visualization techniques to help us understand the nature of fragmentation and opportunities for EHR optimization or redesign.

**Methods:**

Sunburst visualizations capture the EHR navigation structure, showing levels and sublevels of the navigation tree, allowing calculation of a new measure, the Display Fragmentation Index. Time belt visualizations present the sequences of subtasks and allow calculation of proportion per instance, a measure that quantifies task fragmentation. These measures can be used separately or in conjunction to compare EHRs as well as tasks and subtasks in workflows and identify opportunities for reductions in steps and fragmentation. We present an example use of the methods for comparison of 2 different EHR interfaces (commercial and composable) in which subjects apprehend the same patient case.

**Results:**

Screen transitions were substantially reduced for the composable interface (from 43 to 14), whereas clicks (including scrolling) remained similar.

**Conclusions:**

These methods can aid in our understanding of UI needs under complex conditions and tasks to optimize EHR workflows and redesign.

## Introduction

### Background

The ubiquity of electronic health record (EHR) systems has transformed the health care landscape over the past several decades. Yet, even as improved patient care and cost savings have begun to emerge, significant usability impediments have been well documented [1]. One such usability issue for EHRs is display fragmentation, which can be defined as the location of clinical elements or other care-related information on different screens or in different parts of the EHR, or in ways that require searching, scrolling, or other navigation actions to access [2]. Display fragmentation can affect EHR-mediated workflow and the clinician’s ability to analyze patient health information and provide optimal patient care [3].

Research on EHR system usability, including display fragmentation, has often characterized problems at a rather high level of abstraction (eg, violations of usability principles) or in terms of the user’s expression of dissatisfaction. Researchers are beginning to develop new approaches to documenting problems with increasing granularity and specificity as an extension of usability studies [4]. However, few granular methods have been applied to display fragmentation. Thus, the researcher’s ability to understand the impact of display fragmentation on usability, develop potential solutions, and evaluate these solutions is limited.

The work presented in this paper addresses this gap and problem by describing the theoretical background behind display fragmentation and its impact on a clinician’s ability to provide safe and high-quality patient care. It also introduces 2 methods for granularly assessing display fragmentation in health information technology (HIT) systems so that this challenge can be diagnosed, and system redesigns can be proposed.

### Display Fragmentation and Task Fragmentation

Display fragmentation occurs in EHR systems when a user must click through and view many different screens or parts of screens to view all relevant clinical information [2]. This requires sequential viewing and calls for retaining information in memory while other information is sought. This sort of fragmentation may also occur in a densely populated or cluttered screen requiring much in the way of cognitive resources to locate information. Display fragmentation is closely related to and overlaps with 2 other types of fragmentation involved in clinical care. One is *information fragmentation*—the location of important information sources in forms outside the EHR, often in several different modalities such as paper records, faxes that have been scanned to a repository, messages from staff, and even Post-it notes [5]. This type of fragmentation is extremely common in health care, as it is often not possible or desirable for all patient information to be contained merely within the EHR [5]. Processes that predate EHRs and remain operative can determine information location and health professional use of patient information. Information fragmentation can contribute to the deleterious effects of display fragmentation, as information may not be available at the point of care and, as a result, may impair information seeking, clinical reasoning, and the subsequent quality of decision making by health professionals [6,7]. Information and display fragmentation share a core problem that makes it difficult for the health care provider to access needed patient data or pertinent EHR functions.

Although both display fragmentation and information fragmentation involve challenges accessing needed information, their point of emphasis is different. Display fragmentation emphasizes how features of an interface result in a user devoting cognitive resources to interacting with system complexity (eg, unnecessary actions) rather than thoughtful completion of the patient care task. The construct of information fragmentation emphasizes the difficulty of assembling needed information, some of which may be available outside of the system or application, and some of it may rely on the robustness of clinical communication as in patient handoff.

Another form of fragmentation is *task fragmentation*, in which there is a separation of the parts of a task in undesirable ways [6]. For example, the task may be broken into too many steps, or the steps are redundant. This usually slows the overall process of performing tasks using a system, such as an EHR, while at the same time increasing the cognitive load for the user (eg, physician or nurse) performing the task. Undesirable task fragmentation is often a result of display fragmentation and information fragmentation forcing the user to take additional actions to view related material to support their information seeking and decision making. It also fragments the user’s optimal workflow and can lead to workarounds for completing tasks [8]. This is especially the case when new systems introduce new ways of performing cognitive and physical work (eg, to support a therapeutic decision) [9]. This may also be due to other circumstances, including interruptions and the need to reprioritize clinical activities.

HIT systems such as EHRs often create new workflows or can be disruptive to existing workflows, leading to increases in cognitive and physical burdens [3,8,10]. For example, researchers found that the use of a computerized physician order entry system introduces additional steps to view the *patient overview* as compared with the work practices before the implementation [11]. Systems that are not coextensive with clinical workflow may increase the frequency of task switching and multitasking, thereby contributing to a fragmented experience [10]. A recent review of EHR usability and safety literature concluded that navigation is a crucial component of usability [12]. The authors of this paper argue that further usability research is necessary to identify and categorize navigation actions with greater precision [12]. These mapping efforts can provide a uniform approach to EHR usability research and enable systematic comparison between different systems [13].

Research has also shown that reasoning and decision making by clinicians can be highly sensitive to and influenced by the structure and organization of information and information categories in menus and lists, as it is displayed in an EHR system itself [14,15]. The fragmentation of clinical information can create inefficiencies and lead to suboptimal diagnostic reasoning [14,15]. This suggests a need to more closely scrutinize the impact of display fragmentation on clinical cognition. We do this by developing a new method for characterizing fragmentation guided by a cognitive engineering framework. Our approach is interdisciplinary and focuses on the development of methods and tools to assess and guide the design of computerized systems to support human performance [16,17].

### Cognitive Engineering: Characterizing and Visualizing Fragmentation

User interaction can be analyzed as a combination of elementary cognitive, perceptual, and motoric behaviors [18]. All 3 elements are necessary for any task, and specific task-system combinations may be of a more memory-intensive nature or require more in the way of perceptual and motor behavior [19]. Users divide their cognitive resources between navigating through the system interface and performing specific tasks at hand (eg, documenting vital signs) [20]. Seamless navigation is characterized by a fluid interaction in which the effort expended while interacting with the system interface is minimal. Systems of greater navigational complexity necessitate that more effort be devoted to interacting with the system and less to thoughtful task completion [21].

Specific interface elements such as screen layout, pull-down menus, and dialog boxes can affect the levels of optimality or complexity in system interaction [22]. Optimizing the form in which information is displayed, accessed, and documented is dependent on identifying and understanding the flow of specific tasks [21]. Understanding the levels of fragmentation and navigational architecture by mapping specific vendor EHRs can have many applications, including the creation of new navigation tools and streamlining workflows, improving the usability of systems, and decision making. The navigational complexity can be operationalized and measured in terms of the flow or level of interactivity for a given task [21].

This paper describes the methodology behind 2 new approaches for visualizing and quantifying display fragmentation and task fragmentation as they apply to clinician use of EHRs. In the Methods section, we will describe the approaches in detail, including their methodology and examples of their application (titled Illustrations). In the Results section, we will present the results of the illustrations to gain insights into display fragmentation and task fragmentation. The short-term goal of this research, as reflected in this paper, is to show how these methods can provide valuable insight into HIT interface challenges related to display, information, and task fragmentation. The long-term goal is to improve the design of HIT interfaces, such as EHRs, so that they have better fit-to-task, lower cognitive burden, and can enhance clinical decision making, thus improving patient quality of care and safety.

## Methods

### Overview

Two methods used to visualize and characterize display fragmentation and task fragmentation were sunburst diagrams and time belt visualizations, respectively. We first present each of these methods in detail and 3 illustrations that exemplify how each of these methods can be applied to analyze display fragmentation and task fragmentation.

### Method One: Sunburst Diagrams for Describing Display Fragmentation

To understand display fragmentation, we developed *sunburst diagrams* as a method for visualizing system navigation and, subsequently, display fragmentation. Using these diagrams, we developed a measure to quantify display fragmentation and allow easy comparison between systems.

**Figure 1:**
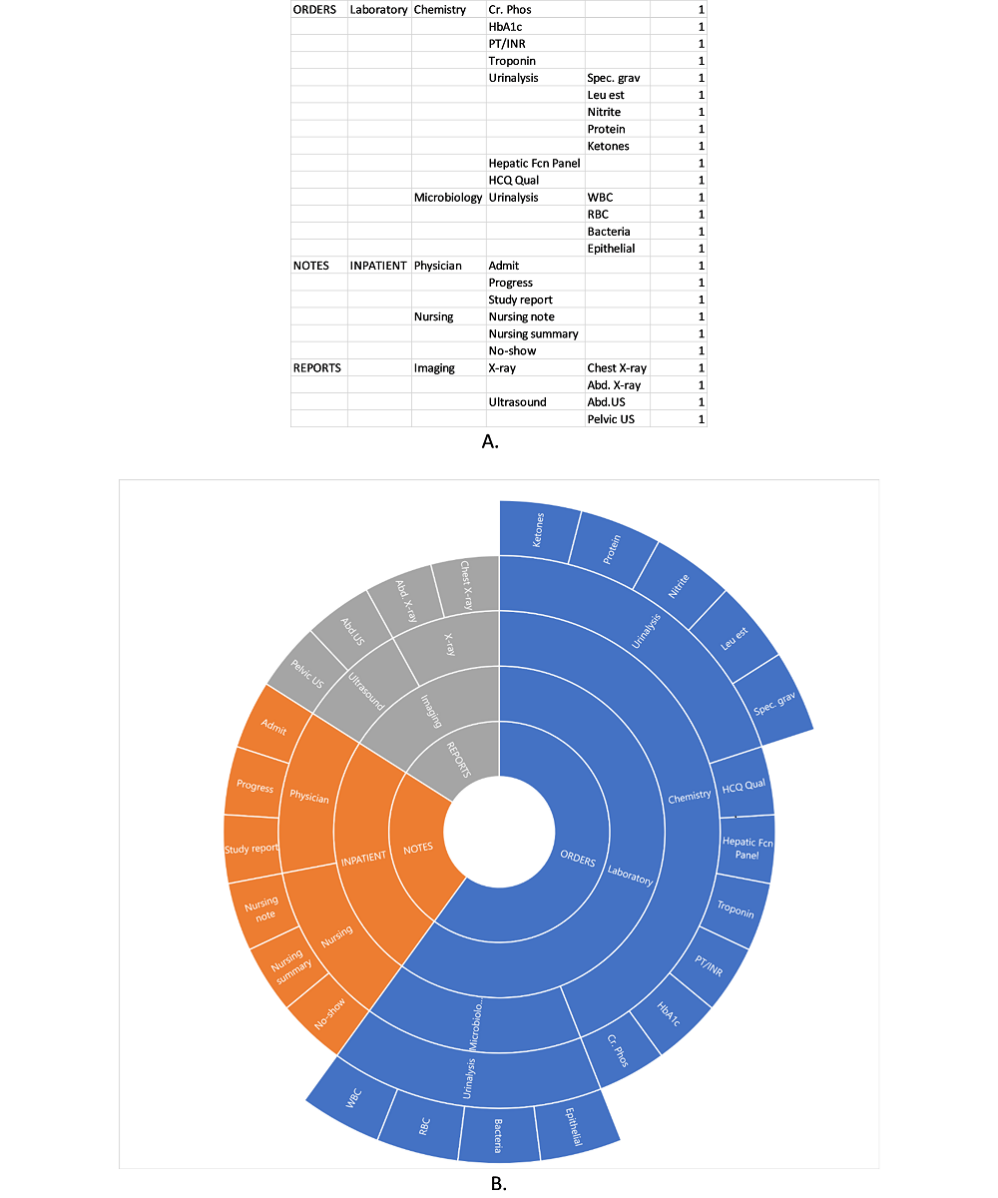
An example of sunburst chart data in Excel describing the system architecture (A) and the resulting sunburst diagram (B).

Navigation in menu-based systems is typically represented in the form of a tree arrangement; on the top-level screen, menus are usually displayed in a left-hand column or as tabs across the top or both. The *root* of the tree represents the highest-level screen, and each level of the tree and its *leaves* represent subsequent menu and submenu choices and varying levels of branching downward. The sunburst diagram presents an alternative, more concise representation of system navigation. The visualization shows the highest level of the system or tree as the first, innermost wrapped circle, and successive levels in the system or tree as successive concentric circles (Figure 1). Screens or levels of navigation that are in the same hierarchical level appear as segments within the same circle. The screens at different hierarchical levels appear as different circles. Therefore, a diagram with a greater number of circles indicates more system levels and screen transitions.

**Figure 2:**
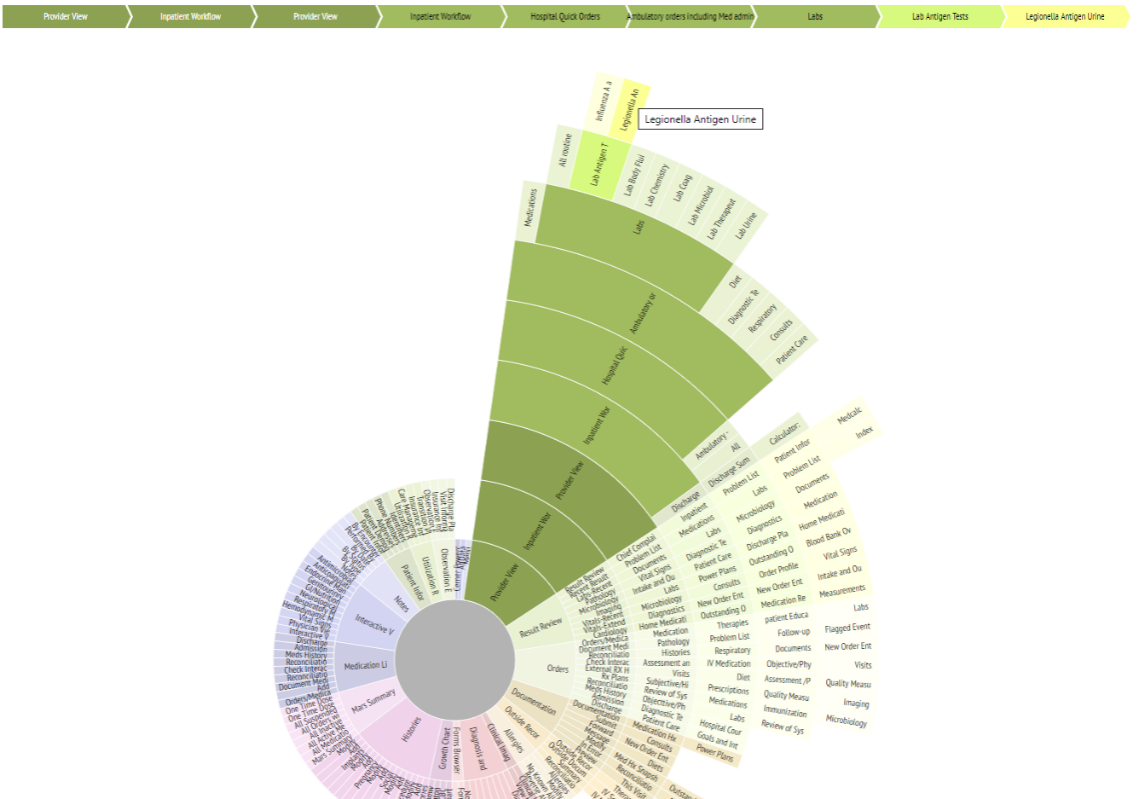
Interactive sunburst highlighting one pathway (in green) from a root screen (Provider View) to a specific element (Legionella Antigen Urine lab results). The traced pathway is also described in the linear flow above the sunburst diagram. This diagram shows how 9 different screens must be navigated to access the desired element from the main screen.

To begin building a sunburst diagram, a modified cognitive walkthrough is performed, in which the researcher steps through all levels of the system’s navigation systematically, recording the menu structures and substructures and how they lead to different clinical data elements or other affordances [15]. This differs from usual cognitive walkthrough methods in that the aim is to create a map of the navigation structure of the EHR, rather than to elucidate the steps needed in the performance of specific tasks. We term this a *modified cognitive walkthrough* to make this distinction. After the modified walkthrough, the recorded information serves as the *data* that populate the sunburst diagram (Figure 1). Excel (Microsoft), for example, has a built-in sunburst diagram function that automatically creates the diagram based on the data. Figure 2 provides another example of a sunburst diagram that highlights a specific pathway.

Sunburst diagrams are advantageous in that, in addition to visualizing the structure and tracing pathways, we are also able to calculate the number of clicks, screen transitions, and other navigation actions needed, such as scrolling or filtering. For example, we can first shade the segments of the diagram that represent target information or screens a specific color (as in Figure 2). Knowing that the transition from one circle in the diagram to another represents a change in screens, and the transition from one segment in a circle to another segment in the same circle represents a click or perhaps screen scroll to view, we can use the sunburst diagram to systematically calculate the number of transitions and navigational actions needed to navigate from one target piece of information to another. We used this benefit of the sunburst diagram to create a measure to quantify display fragmentation and navigational complexity, which provides a basis for comparison between systems or between tasks. We termed this measure Display Fragmentation Index (DFI).

**Figure 3:**
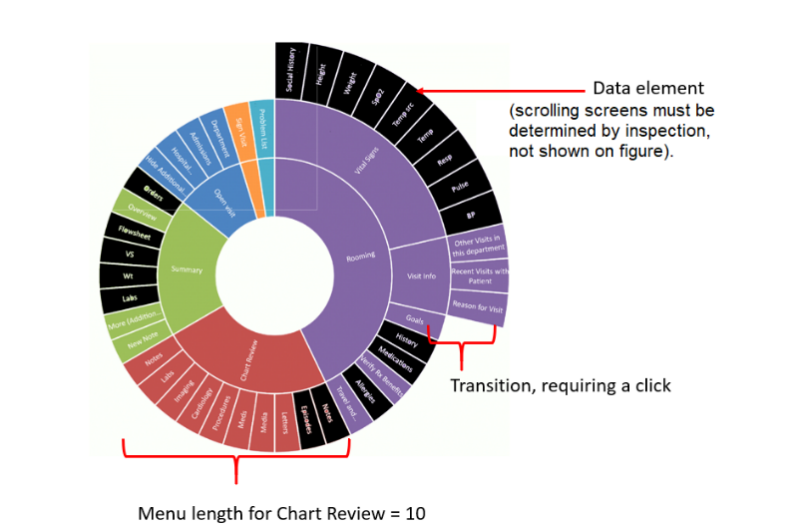
Display Fragmentation Index element calculations. DFI: Display Fragmentation Index.

The DFI captures (as in Figure 3): the overall number of different content categories into which the required information is split; the different levels of the tree structure, with each level requiring additional clicks; navigation to elements at the same level, which also requires at least one click or scroll action (with multiple scroll actions counted as 2, as an average owing to the variability of such screens across cases); and the menu length (parallel items, which appear adjacently) at each stage, as this reflects the complexity of choice (and hence a taxing cognitive task) among menu items and results in greater visual search. Menu length is also highly indicative of the navigation time [23].

Thus, we can calculate DFI using the following equation:

DFI = E + IS(X_IS_) + C(X_c_) + SS(X_ss_) + ML

where E refers to the number of data elements, IS the number of intermediate screens (transitions), C clicks (navigation action), SS scrolling screens (navigation action), ML menu length, and X a multiplier applied to each variable based on the number of levels traversed.

E does not have a multiplier because it simply represents the number of information elements that need to be accessed, regardless of their location.

To calculate this measure, we focus on the main obvious navigation paths as the measured pathway. As many EHRs may have several routes to get to an item, the fragmentation measure is a reasonable maximum; for some tasks, one may not have to go up all levels to get to the next item, as just the lower levels may be involved. The actual trajectory may vary depending on the user’s goals and preferences; the one presented is the longest reasonable pathway.

In this illustration, we show how sunburst diagrams allow easy comparison of fragmentation and navigational complexity between systems and can be used to calculate DFI.

### Illustration Overview: Using Sunburst Diagrams to Describe Display Fragmentation in EHRs and Developing a Measure to Quantify Display Fragmentation

To show how sunburst diagrams can be used to describe and quantify display fragmentation, we illustrate how we used sunburst diagrams to visualize display fragmentation for 2 different, widely used, commercial EHRs. The context of this illustration is that the research team sought to understand the extent of display fragmentation in commercial EHRs, including differences in navigational architecture. Thus, the team conducted a modified cognitive walkthrough and used the sunburst diagram to display the results.

The team then conducted a more traditional, task-oriented cognitive walkthrough emulating the process of clinicians conducting general case reviews. This is a second step in the method, after creating the general navigation map described in the Method One section above. Data and information types for this task included admission notes, laboratory results, orders, medications, allergies, study reports (eg, of imaging or other studies), images (if available), discharge summaries, primary care and specialist provider notes, medications, demographic and insurance data, and nursing notes (if available), and automated data from devices or mobile apps, if applicable. Most current EHRs house similar data types together, necessitating complex navigation to see all relevant types while evaluating a patient case.

Once the diagram was created, the number of screen transitions and levels of navigation were counted using the visualization, and DFI was calculated.

### Method Two: Time Belts for Mapping User Workflow and Task Fragmentation

We also developed a method for visually characterizing user workflow and task fragmentation. Visualization methods provide a systematic way of graphically representing information in a way that allows for understanding work and cognitive processes. Cognitive visualization methods allow for the use of visual metaphors for gaining insights into user mental steps and mapping of user workflow. Many of these methods are linear, but there are nonlinear metaphors as well, such as the desktop, tree, or swimlane metaphors. Understanding how EHR navigational structure affects workflow can be aided by additional mapping of the user’s actions while performing a task.

**Figure 4:**
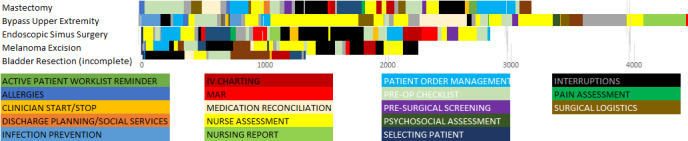
An example of time belt visualization for 5 patient cases in preoperative care at a large tertiary care hospital. One single horizontal belt or row represents 1 patient case. The length of the belt indicates the case duration in seconds. Each belt comprised a sequence of tasks performed by the nurse and represented as color-coded segments. For example, Allergies refers to the task of checking allergies.

Time belt visualizations involve the linear depiction of the different phases and actions of a user (Figure 4). The different information types viewed, durations, and repetitious navigation to the same elements or element types can all be conveyed succinctly to understand a user’s work patterns. In the diagrams, the percentage of time shown in each section of the system is easily identified by a key, and the time sequence of the user is clearly shown from the start of interaction with a system to the completion of a task. Such an approach can be used to graphically depict an individual user’s patterns in accessing components of an EHR over time for comparison purposes (eg, comparing residents vs attending physician interactions with cases of differing complexity). Zheng et al [24] investigated the variation in preoperative workflow findings in 2 hospitals. Suboptimal patterns were identified, and the reasons for the variation were explored. Although both settings used the same EHR system, they observed marked differences in patterns of workflow with consequences for patient care. Figure 4 shows an example of a time belt that represents workflow as a series of discrete tasks representing their sequence (color-coded) and their duration (width of the colored segment). A simple representation can be used to compare clinicians, EHRs, patient conditions, and visit types. The time belt reflects the overall flow across the patient’s encounter. We can also drill down to examine the navigational complexity for specific tasks, such as medication reconciliation.

Time belts can be used to compare time on tasks across different systems, as well as the time spent on different tasks. This representation enables us to scrutinize task performance at a granular level, including time spent on different tasks, fragmentation in terms of repeated tasks, and sequential ordering of tasks. We can also examine each segment and determine the degree of interactivity. Importantly, we can break down a task and characterize clinicians’ clinical reasoning and, specifically, how diagnostic and therapeutic reasoning evolves over the course of time.

We also calculated the proportion per instance. Zheng et al [24] derived a measure of task fragmentation that relates the time on task for subtasks, normalized for EHR time. Their measure, time proportion per instance can be used to compare EHR tasks in different settings:

Proportion per instance = Instance task time/Number of task instances × Total EHR time

This is based on average continuous time (ACT) in which increased task fragmentation results in decreased time for a subtask [3]. The longer the ACT, the lower the task fragmentation. The proportion per instance normalizes this to accommodate total EHR time so that longer sessions do not inflate the measure.

### Illustration Overview: Using Time Belts to Compare Task Fragmentation and Workflow for a Conventional EHR and a Composable EHR

We developed an experimental EHR interface to address some of the issues of fragmentation and cognitive load [25-27]. The following illustration details how the described visualization techniques were used to compare a commercial EHR with the experimental system.

The context for this illustration was a larger study where medical residents were recruited and presented with a series of cases using real patient data in a conventional EHR or the experimental system. The patient data were collected at a large health care site as part of a larger study examining EHR-mediated nursing workflow. For each of the cases, the patients were *seen* by other clinicians previously, and the study participants were asked to review the documentation, determine the reason for the patient’s problem, and present a therapeutic and management plan of action. The participants’ interactions with the systems were captured by Morae (TechSmith) [28], a powerful video recording and analytics tool widely used in human-computer interaction research. Participants were also asked to think-aloud while completing the tasks, and their dialogue was recorded and transcribed.

After the study, the recordings were analyzed, and time belt visualizations were created to compare time on tasks across the conventional and experimental systems. Note that this illustration is meant to show how time belt visualizations can be used to surface different dimensions of clinical cognition. It is not intended to compare the efficacy of the conventional and experimental systems, but rather exemplify how their interfaces yielded different patterns of interaction.

For the purpose of this illustration, we present the results for one resident participant who used the conventional EHR system to examine a patient case, John Smith, and a second participant who used the experimental system to examine the same patient. In the scenario, John Smith had an extensive medical history and presented with an array of cardiac and other clinical problems. He is in the emergency department (ED) due to exertional chest pain starting 2 hours previously (severe, 10/10, sharp or stabbing, localized as substernal, radiating to the back). The clinician is an ED physician treating the patient and has some past EHR records, including 2 prior progress notes and medical or surgical history, laboratory values, allergies, social history, and medications.

### Illustration Overview: Putting It All Together—Sunburst and Time Belt Visualizations

We present a third and final illustration in which we show the 2 visualization methods in tandem. The context for this illustration is similar to the one for the time belts: a larger study where medical residents were recruited and presented with a series of patient cases that used anonymized but real patient data in the conventional EHR or experimental system.

Participant interaction with the system was recorded and then used to create a time belt to show the time spent on each screen or element. Similarly, the EHR was mapped using the sunburst diagram, and the resulting Excel sheet was used to show how the user participant navigated across the system.

## Results

### Illustration Results: Using Sunburst Diagrams to Describe Display Fragmentation for an EHR System and Developing a Method to Quantify Display Fragmentation

**Figure 5:**
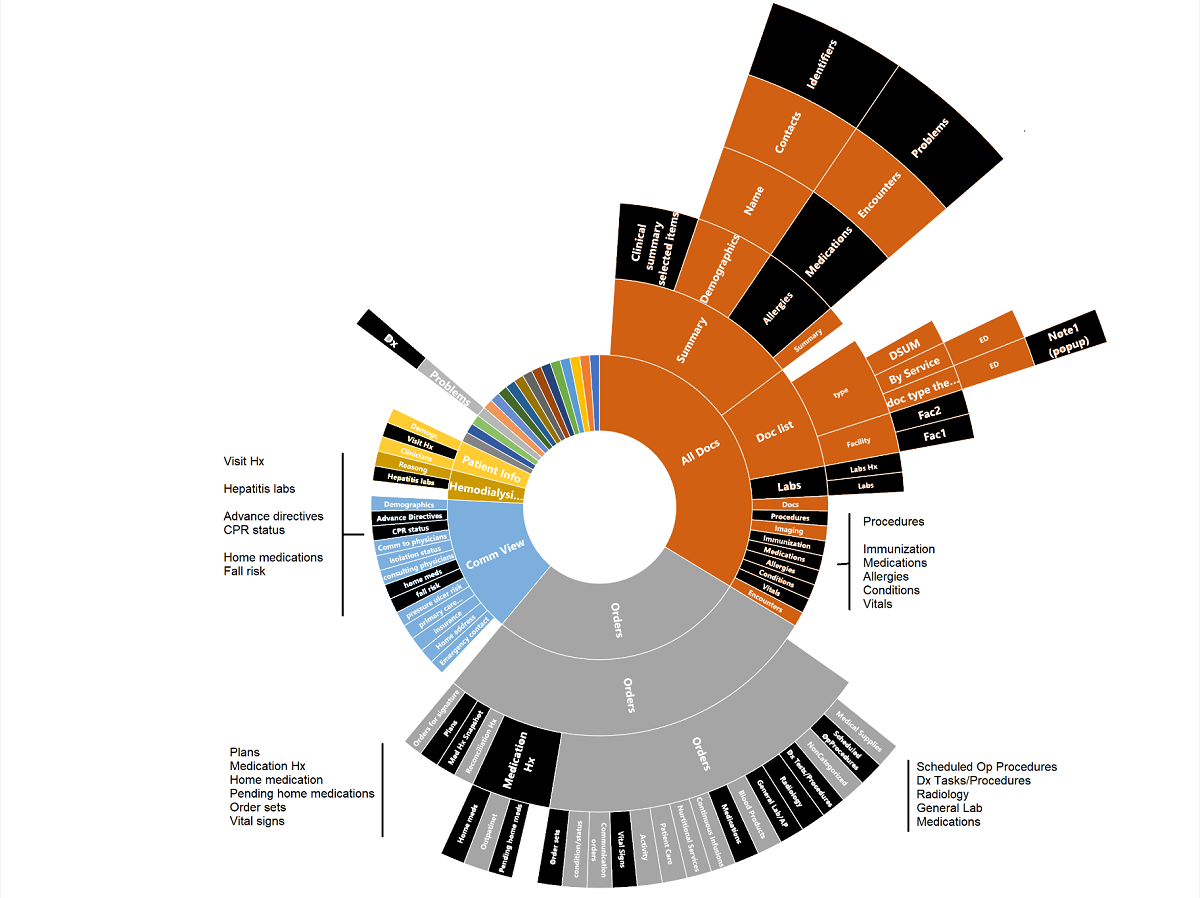
Sunburst diagram representing display fragmentation of clinical data in a conventional, commercial electronic health record. Elements colored in black are those relevant for handling the clinical problem (general review of patient information). EHR: electronic health record; UI: user interface.

The sunburst diagram for the cognitive walkthrough of the first conventional EHR is presented in Figure 5. The elements colored in black represent the information relevant to the clinical task used for the cognitive walkthrough—general case review. One can see how the relevant information elements are scattered across different paths, levels, and main sections.

Using the DFI measure described above, the DFI for the sunburst diagram presented in Figure 5 was calculated as follows:

DFI = 36 + 136 + 136 + 39 = 347

Note there are no scrolling screen terms incorporated into the above equation.

Thus, it is easy to conclude that the degree of fragmentation for this conventional commercial EHR is rather high and could likely lead clinicians to spend a large amount of time and cognitive resources while navigating, viewing, and retaining information.

**Figure 6:**
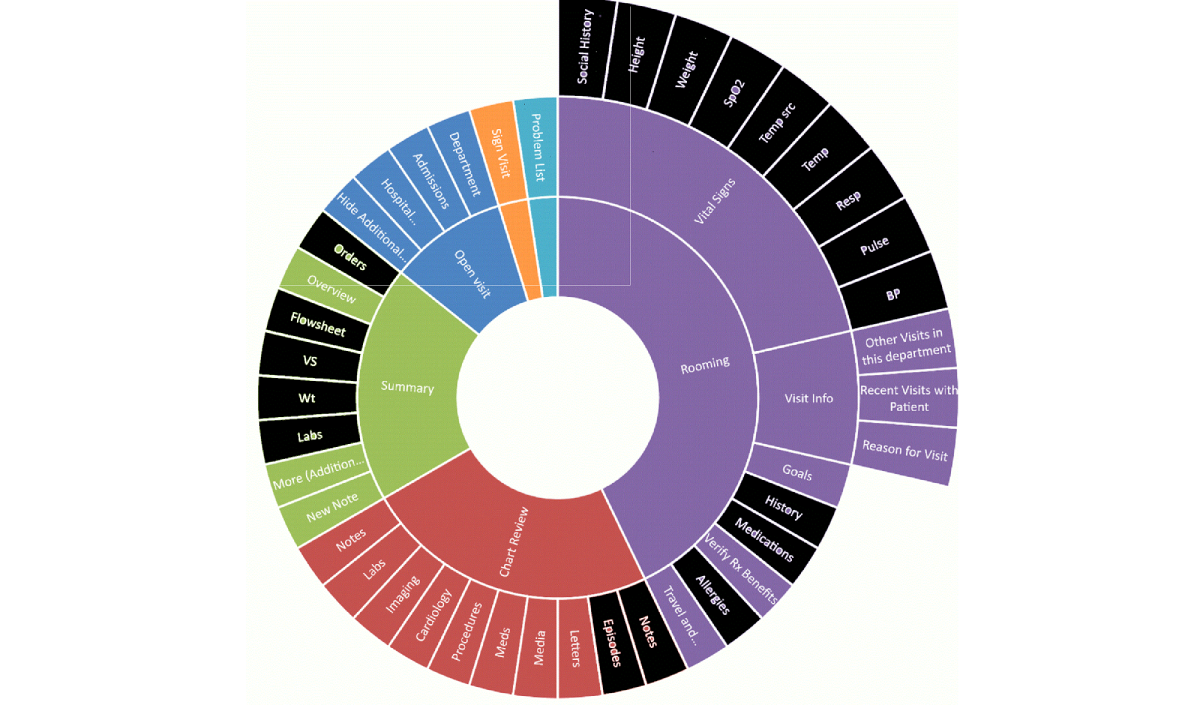
Sunburst diagram representing display fragmentation of clinical data in a second conventional, commercial electronic health record. Elements colored in black are those relevant for handling the clinical problem (general review of patient information). EHR: electronic health record.

The researchers also analyzed a second commercial EHR user interface (UI); the resulting sunburst diagram is presented in Figure 6. For this system, the DFI was calculated as follows:

DFI = 19 + 47 + 47 + 19 = 132 (again, with no scrolling screens term)

Thus, this system has a lower DFI (approximately one-third of the previous system), representing less fragmentation and fewer navigation levels.

### Illustration Results: Using Time Belts to Compare Task Fragmentation and Workflow for a Conventional EHR and a Composable EHR

**Figure 7:**
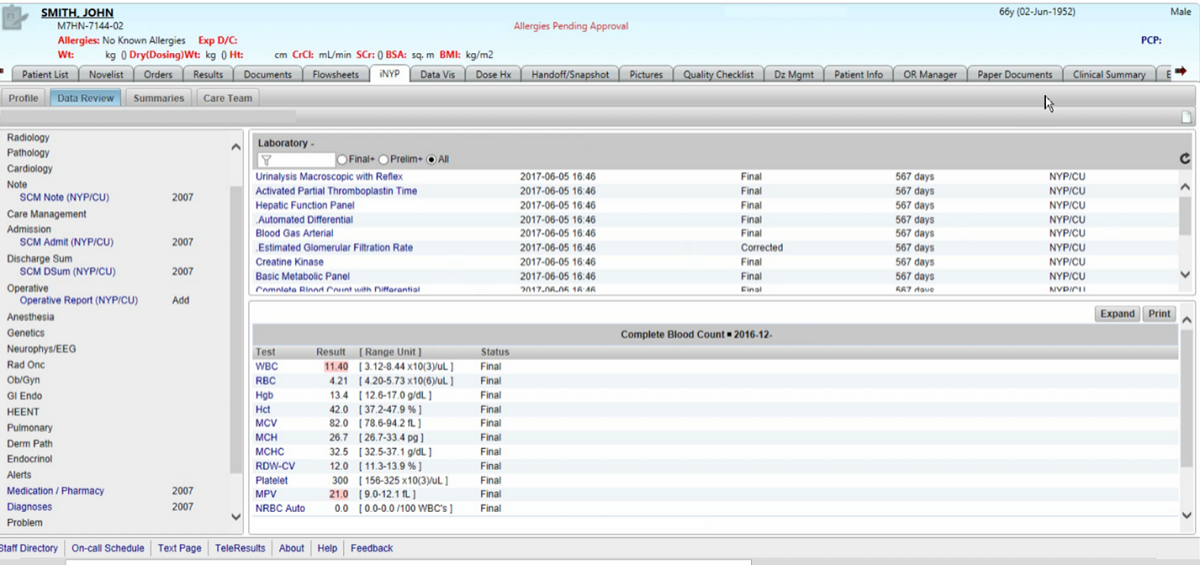
Conventional electronic health record (EHR) system user interface (UI).

Figure 7 presents a captured screen from the conventional EHR system presenting laboratory results for the patient case, John Smith. In the conventional EHR UI, information is accessible through a hierarchical set of tabs, menus, and side panels. To its credit, the conventional EHR UI is well segregated and organized. Much of the patient information can be accessed through the display. On the other hand, the interaction space is immensely complex, and there are multiple ways to access the same information.

The participant used 346 mouse clicks, including just under 200 left-mouse clicks. In that short span of time, the resident visited 43 display screens, including repeat visits to several displays (eg, blood gas arterial panel). She experienced some difficulty locating an appropriate index document, such as a progress note or discharge summary. As a consequence, the resident devoted considerable time to searching for information. She focused largely on laboratory values, some of which seemed anomalous or contradictory, and then toward the end of the session, came across 2 ambulatory care text documents (at the 200-second mark) that facilitated her development of a complete patient problem representation.

**Figure 8:**
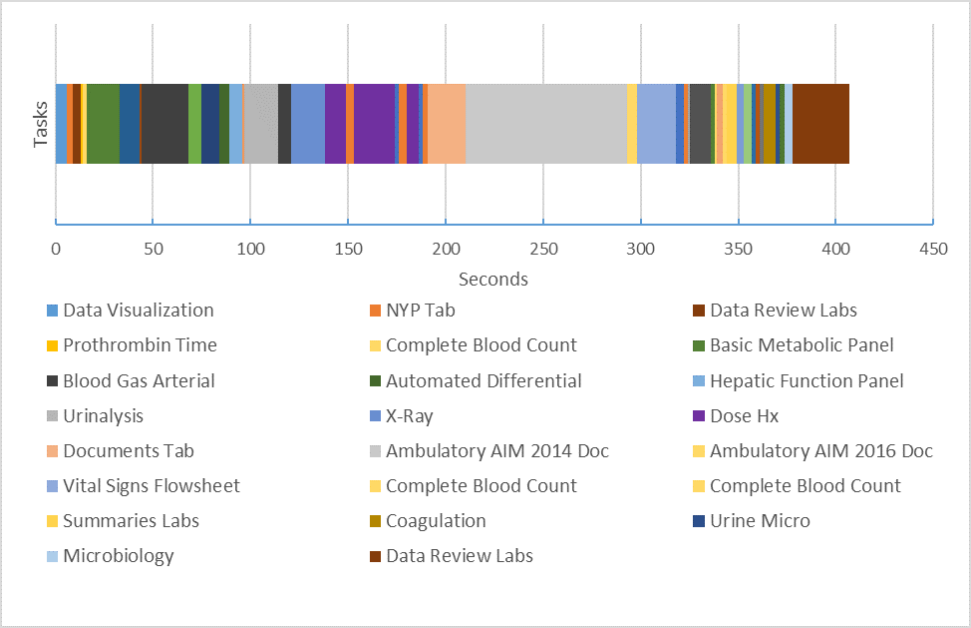
Time belt visualization of clinical task using commercial electronic health record. Note that the labels for the tasks have been abbreviated for readability. Each item represents a task, primarily searching and reviewing tasks. For example, “X-Ray” is short for “Reviewing X-Ray,” a task the clinician completed.

Figure 8 presents the time belt that illustrates the workflow or time on task for the single resident participant performing the task on the conventional EHR system. The participant required 6 min and 35 seconds to complete the task. The time belt is divided into task segments of variable durations.

**Figure 9:**
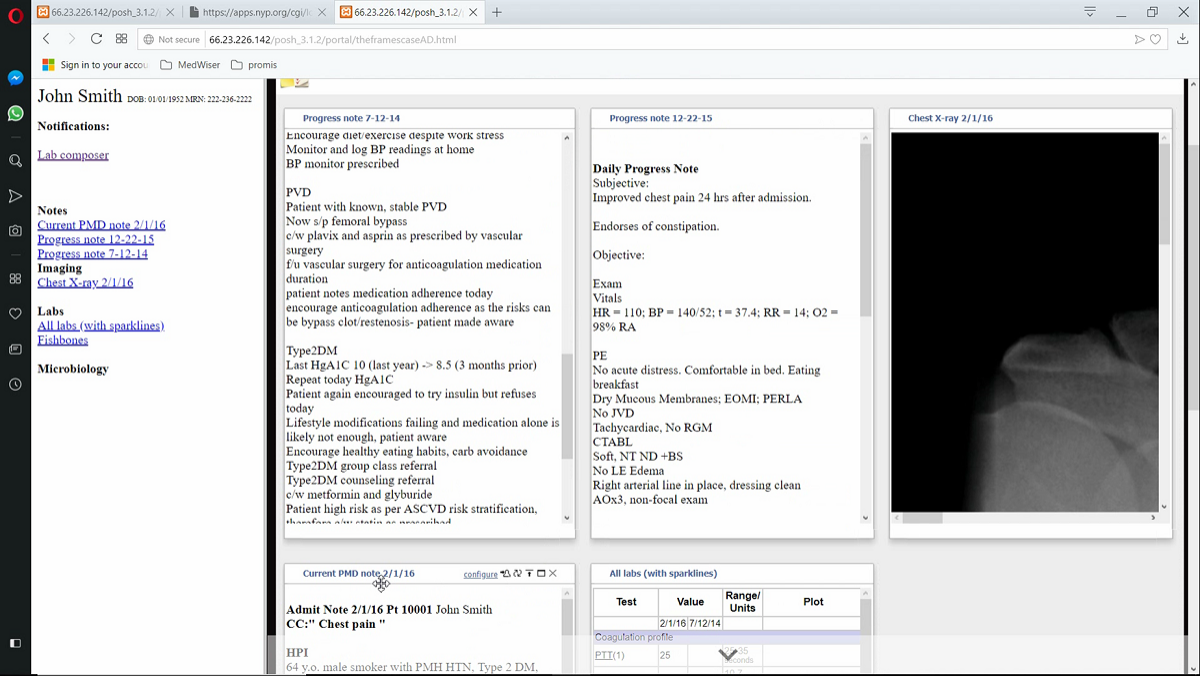
Experimental system screen with user placement of data elements for the same case as in Figure 8.

Figure 9 presents the experimental system interface for the same case. The interface is entirely configurable. The left-hand panel contains a set of available documents relevant to the case. There are only 7 documents, including the contemporary (current) note, 2 older progress notes, a chest x-ray, labs, and Fishbones. Users can drag and drop documents and rearrange them accordingly. The screen below includes 2 rows of documents in the form of widgets. The first row contains 2 older progress notes and an x-ray. The bottom row includes the current document and all laboratory values.

**Figure 10:**
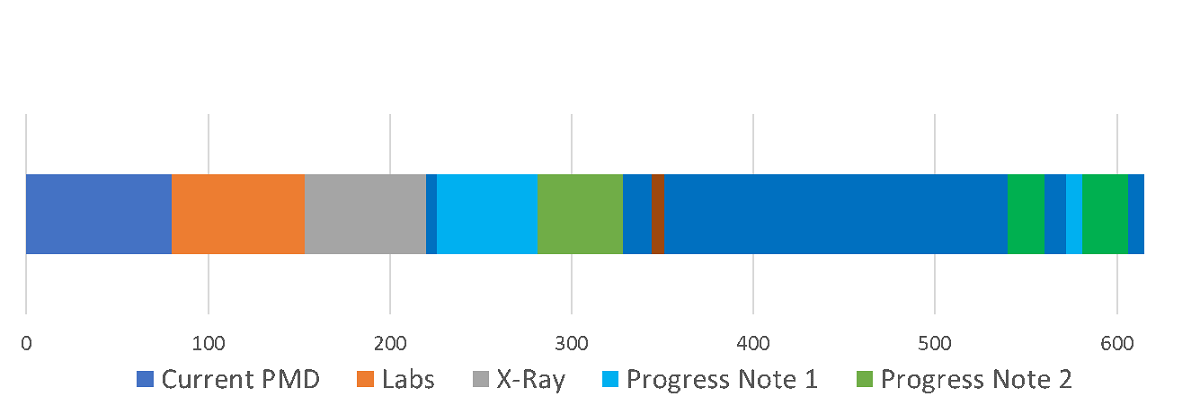
Time belt visualization of clinical tasks using experimental electronic health record (EHR) system.

Figure 10 illustrates the workflow or time on task for the resident performing the task using the experimental system. The task required 10 min and 48 seconds to complete. The user employed 389 mouse clicks, including only 20 left-mouse clicks. The remaining clicks reflect the extensive use of the scroll wheel. In that short span of time, the resident visited 14 display screens, including repeat visits to the current note and older progress notes. The current note acts as the index document to understand the patient’s problem. As we can see, the time belt and other visualizations can be used to characterize a state of affairs—the current state of navigational complexity and fragmentation. They can also be used as guideposts for design at a granular level of interactive behavior.

As noted, participants’ think-aloud statements were recorded. Multimedia Appendix 1 presents a summary of the participants’ think-aloud statements as it relates to the time belt presented in Figure 10.

### Illustration Results: Putting It All Together—Sunburst and Time Belt Visualizations

**Figure 11:**
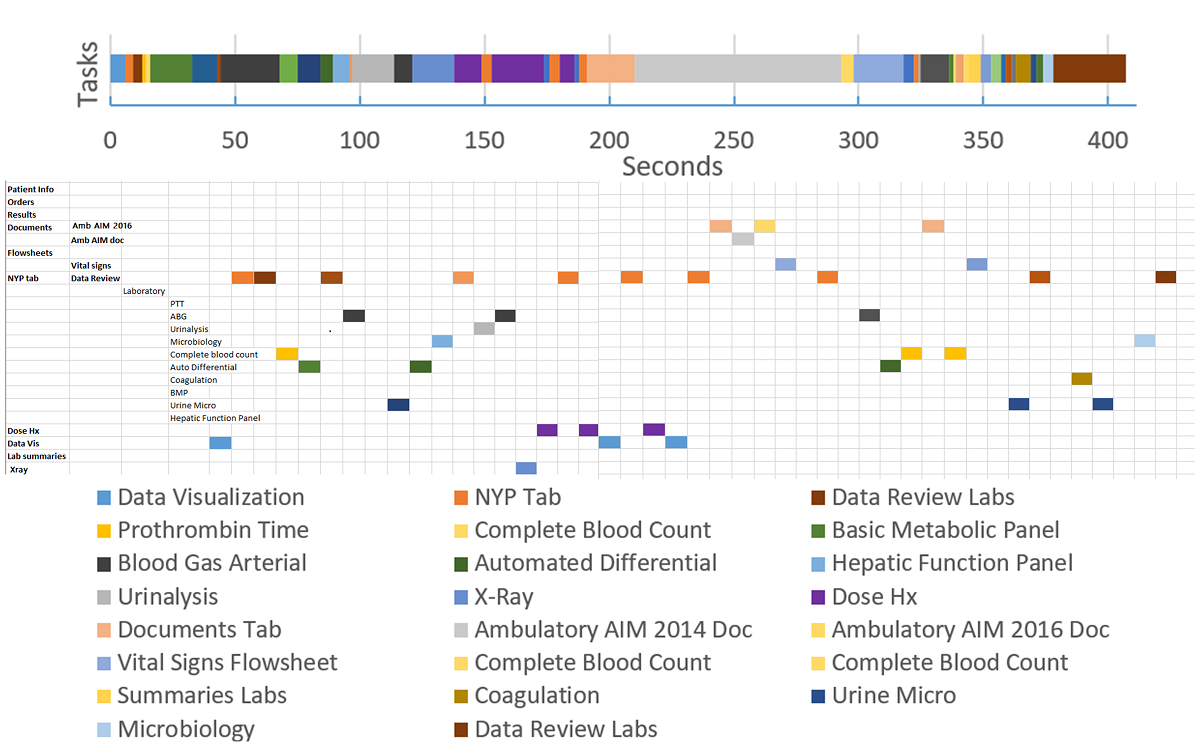
Color of each cell or segment represents the task or screen the user was completing in sequential order. The time belt visualization shows the time taken for each task, whereas the Excel data scheme shows the different screen or part of the system needed for each task.

Figure 11 shows the time belt and sunburst diagram data for a user completing a patient review using a conventional, commercial EHR. The time belt shows the time spent on each task or screen. The Excel sheet shows the data for the sunburst diagram; however, in this instance, cells have been shaded to show the order and pathway the user took to navigate the system. For example, the user started on the *Data Visualization Screen* (light blue), then navigated to the *NYP Tab* (orange), then navigated to *Data Review Labs* (brown) within that tab, then *Complete Blood Count* (yellow), and so on and so forth.

From this combination of the time belt visualization and the sunburst diagram data scheme, we can see how task fragmentation corresponds with display fragmentation. Researchers can easily deduce how the user becomes *stuck* in several instances of back and forth navigation between 2 screens, and viewing sequences involving items far apart (see the number of orange-shaded cells indicating the user visited *NYP Tab*).

An example of proportion per instance is provided in Figure 11 time belt. Note that a lower proportion per instance value denotes more fragmented subtasks per unit time. In the time belt, this shows as a higher number of bands of the color corresponding to the task instances in a single patient encounter.

**Figure 12:**
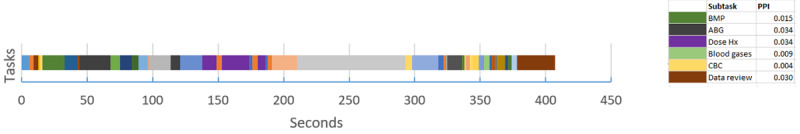
An example of proportion per instance calculated on the basis of the time belt from Figure 11. Proportion per instance for certain subtasks have been calculated in the small table to the right as examples. PPI: proportion per instance.

An example use of the measures can be seen to optimize displays by reducing fragmentation. In the time belt of Figure 12, the period from 140 to 200 seconds was spent in back and forth navigation looking at the same 2 elements 3 times each, with a navigation action screen between. Optimization could consist of juxtaposing these 2 elements, reducing the need for back and forth navigation. The proportion per instance would be improved for the same task.

## Discussion

### Principal Findings

This paper illustrates a set of methods and visualizations to characterize navigation complexity. It is based on the analytic work and video capture of users. There is a range of methodologies that can further inform our understanding of the problem. For example, eye movement studies have the potential to elucidate the relationship between the visual apprehension of information and clinical reasoning [4,29-31]. It should also be noted that EHR-mediated workflow extends beyond the confines of navigational complexity to a host of other issues necessitating convergent methodologies [32-36]. The work presented in this paper is formative and is part of a growing body of theoretically motivated research that seeks to expand the vision of usability and better situate it in the context of clinical workflow and quantifying complexity [20,21,24,37].

The sunburst diagram can serve many cognitive science, usability, and workflow analysis purposes. First, a static sunburst diagram is useful in simply providing a visual representation of the current system state regarding navigational complexity—it shows the structure of the system. The diagram can also be used dynamically and interactively to show the influence of redesign decisions on navigational complexity or screen fragmentation. Interactive versions permit clicking on a segment, which will then be shown as the top (the inner circle) of the resulting subtree, with subsections as concentric circles (Figure 2). This can then permit extensive drill-down and visualization of the entire tree even if it is very complex.

Second, the sunburst diagram permits viewing of the relationships between different parts of the system and facilitates the tracing of navigation pathways through the system (Figure 2). Once created, viewers can easily trace pathways for access to a certain system element or screen and thus further characterize display fragmentation. Pathway tracing and navigation map building are completed during a cognitive walkthrough [38], as described above. In web-based systems, there are web tools that can automate this process (eg, Powermapper [39] and edraw [40]), but many major vendor systems are not web-based. Furthermore, greater insight can be gained by differentially coloring the segments of the diagram, which can help to further visualize the degree of fragmentation. For example, one color can denote the data of interest, and another color can denote irrelevant data for a task. Thus, someone can easily see the proportion of useful information to extraneous information and take action to redesign the system to remove the extraneous information.

The sunburst and time belt diagrams are complementary visualizations. The time belt is a succinct and clearly understood representation. One can view the distribution of tasks more readily. The time belt has a more explicit temporal dimension, which makes it easier to make inferences about the distribution of time.

Clinical care presents many pressures to the clinician; these are increased in emergency settings and where cases are complex. Current EHR systems interrupt clinical reasoning and workflow, increasing these pressures. The ideal system would rapidly present salient information and data critical to decision making and mitigate clinician cognitive load. We have found in studies of composed displays (where this is the case) that subjects find that the lack of the usual interruptions due to excessive navigation is cognitively supportive and helps their thought process. The methods described here can help to build such systems.

Ultimately, our aim is to make the arduous and already difficult work of clinical care smoother, more accurate, less cognitively demanding, and more pleasant. Ideally, information tools should be transparent, *fun to use*, enabling user control and freedom, and permitting focus on the tasks at hand rather than the tools themselves. Findings from our formative experiments suggest that composed displays that minimize the need for navigation can have this effect (prelim work, Y. Senathirajah et al, unpublished data, 2021). Some tool use in other domains approaches the level of artistic integration between users and tools to accomplish the most difficult of tasks. Although we are far from this in HIT, perhaps diligent further work can bring us close to the aim in the future. Consumer tools (such as some Apple products) have been studied for this quality of pleasure in use. Many clinicians view EHR use as unpleasant and unsatisfactory [41]. Reducing navigational complexity and facilitating task performance may free the clinician to be more creative, resulting in a more productive and pleasurable experience.

Although EHR usability has been much criticized, there is a very large installed base of the current EHR software. In conducting EHR mapping and task microanalysis, we aim to move beyond static conventional usability testing and bring together usability information with very particular guideposts, provide opportunities for EHR optimization, and more generally HIT redesign. A distinguishing feature of the composable EHR approach is its ability to juxtapose elements to decrease navigational complexity (thus increasing display integration). Understanding how the current EHR structure imposes fragmentation on both information access and task performance opens the way for specific focused redesign, which could shorten navigational pathways and thus time and effort taken. Decreasing screen fragmentation decreases the load on working memory. It could also permit specialized displays with low cognitive burden and machine delivery of UIs optimized for tasks.

We have derived useful measures addressing different needs for the comparison of EHR structure and its effect on navigation and task performance fragmentation. Having DFI and Zheng’s proportion per instance permits making a distinction between different EHRs or their subsections for the same task, and different tasks carried in the same EHR as well as in different locations, by different clinician roles, and other factors. DFI can be used to distinguish EHR structure and navigation, subtasks for different EHRs, subtask time efficiency, and EHR interface redesign. Proportion per instance can be used to distinguish tasks, subtasks for different EHRs, subtasks for the same EHR but different clinician groups, and subtask time efficiency.

Subtask time efficiency is an important measure for finding areas in which EHRs can be optimized. Although DFI has no time elements, by identifying areas of fragmentation in navigation structure and therefore likely in task performance, it can aid in finding areas in which *pogo-sticking*, that is, navigating back and forth between elements or sections of the EHR occurs or is likely to occur. When this occurs repeatedly (eg, when a clinician reads a note and switches back and forth repeatedly from the note to a lab values section to check the current values of laboratory tests against those listed in the previous note), it is a subtask. The juxtaposition of the two elements (note and laboratory test results) would avoid this repetitious navigation, shortening the subtask time, and removing the excess navigation or clicks. The juxtaposition is known to foster reduced cognitive load (as data need not be retained in working memory as it is on screen together), reflection, the association of data elements, and the identification of patterns. In the note or labs example, the user will be able to see the change in laboratory values and the implications of such changes more easily. Providing both better cognitive support and shorter times or less navigation would aid in reducing the burden on clinicians, particularly in high-stress settings. EHRs are heavily implicated in contributing to physician burnout primarily because of the mismatch between task and system, leading to poor efficiency and frustrating navigational complexity.

Finally, we address the experimental system used in our illustrations. MedWISER is a system in which elements are easily arranged by drag or drop. Therefore, we can design novel UIs using MedWISER using the same elements used in conventional system tasks (eg, lab panels) to represent data and experiment with different configurations with the intent to simplify navigation. In the above subtask example, the clinician user can juxtapose the note and lab together by drag or drop without requiring programmer intervention; this is a normal way the system functions. Thus, the end user, or others such as researchers or system administrators, can easily rearrange data elements to foster shorter navigation paths, the juxtaposition of related elements, and the creation of screens that maximize support of clinical reasoning while minimizing excess navigation. The user’s arrangements are stored, and patient-specific or specialty-specific displays can be shared (eg, with colleagues taking the next shift for that patient; they can also further modify the patient-specific display as new data comes in), further minimizing excess navigation and multiplying time savings. Thus, a set of displays could eventually be created with minimal fragmentation for the tasks being done (as can be calculated with new displays using our measures to evaluate degrees of optimization).

### Limitations

There are several limitations to this study. Our presentation of cases is illustrative of the methods and is not representative of a larger set, which should perhaps be a next step in validating the work and formulae. Although the representations surface important dimensions of workflow, the comparative illustration of different systems cannot be used to infer that one system is better than another. There are many ways to represent data, and we have chosen ones that enable us to draw inferences about fragmentation based on a set of observations. The goal is also to convey the information visually and reliably so that readers can readily draw the same inferences or alternatively draw their own conclusions. However, there are other visualizations that may convey the same information. It is difficult to definitively prove that sunbursts or time belts are superior to other forms of representation. Expanding the space of potential visualizations can help advance the study of EHR-mediated workflow and, perhaps, its communication to stakeholders beyond academia.

### Conclusions

In this paper, we described a methodological approach to addressing display fragmentation. Novel visualizations provide a suite of tools for communicating the exact nature of a navigation problem and creating the potential for precise and measurable design solutions. EHRs still present formidable usability challenges, but the potential for small tractable changes rather than large-scale prohibitively expensive ones is increasingly realizable. When combined with platforms such as MedWISER, which reduce the work involved in reconfiguration, they could provide pathways for rapid usability improvements and significantly improved the EHR-mediated workflow.

## Appendix

Multimedia Appendix 1 Participant think-aloud process.

## Abbreviations

ACT: average continuous time
DFI: Display Fragmentation Index
ED: emergency department
EHR: electronic health record
HIT: health information technology
UI: user interface
