# Supplementary material for: Characterizing and Visualizing Display and Task Fragmentation in the Electronic Health Record: Mixed Methods Design
Source: JMIR Hum Factors. 2020 Oct 21;7(4):e18484. doi: 10.2196/18484 (PMC7641790; doi:10.2196/18484)
Supplement: Multimedia Appendix 1 [file humanfactors_v7i4e18484_app1.docx]

**Supplemental Appendix I**

The following presents excerpts from the participant’s think-aloud process as presented in the main body of the article.

The following excerpt from the participant’s think-aloud recording corresponds to the first segment of the timebelt presented in Figure 7, which lasts about 80 seconds:

*“OK so he was presenting for chest pain, 64-year-old, smoker, hypertension, diabetes, peripheral vascular disease, coronary artery disease, stress with PCI in the past. Sounds like he's a lot of vascular comorbidities. Yikes. GERD, chest pain two hours before presentation. Again, this is in the past there. Right. OK. Went to the ED. Already on aspirin at home, Simvastatin, Metoprolol, Lisinopril for his heart disease, Metformin and Glyburide for his diabetes, smoker… that time he was quite tachycardic and hypertensive and a little bit hypoxic as well. Seem to be in some distress. Nothing too revealing on physical exam.”*

She subsequently selected the lab panel, which revealed:

*“A1C is elevated then in the past…in the past seems like phosphorus was low. That was kind of the bigger the most important value there. OK so sounds like he had chest pain but the troponin was negative.”*

The third display, the X-ray revealed that the patient had “sternal wires” indicative of surgery, but not much else. The two subsequent displays constitute the two previous progress reports. They reveal that the patient had GERD, a femoral bypass and an aortic dissection. For the remaining six minutes of the session, the resident juxtaposes the various documents, arranges and rearranges them to fully comprehend the patient problem and to develop a relatively comprehensive management plan. The excerpt below illustrates part of that plan.

*“[00:34:25] Okay so for his hypertension and I sort of put in a lot of the vascular things into the same category. So, for instance, a coronary artery disease, status post PCI, but then also the Aortic Dissection can be really important today to evaluate what his current symptoms are. Particularly if he has chest pain either at rest or with any exertion. What his exercise tolerance is. [00:35:03] And then I like to also ask all the heart failure questions so does he have any lower extremity swelling. Can he lie flat at night those kinds of things. And then if he endorses any of those things, I would be worried that he was having either stable angina or unstable angina and would have to get labs like enzymes and EKG to see if anything active is going on. But presuming that he's not coming in with active chest pain, I would be trying to optimize his medication regimen which would probably include up-titration of his metoprolol to max dose as tolerated by his vital signs.”*
